# The impact of mental fatigue on repeated sprint and change-of-direction performance in soccer

**DOI:** 10.3389/fpsyg.2026.1842345

**Published:** 2026-05-13

**Authors:** Wei Yang, Ting Chen, Gong Chen

**Affiliations:** 1 Department of Physical Education, Quzhou University, Quzhou, China; 2 Post-Doctoral Station of Sport Science, Fujian Normal University, Fuzhou, China; 3 School of Athletic Performance, Shanghai University of Sport, Shanghai, China; 4 College of Teacher Education, Quzhou University, Quzhou, China

**Keywords:** cognitive fatigue, mental fatigue, physical performance, soccer, sport

## Abstract

**Purpose:**

Elucidating the impact of mental fatigue (MF) on physical performance in soccer is essential for optimizing athlete assessment, training, and competition strategies. However, the relationship between MF and both repeated sprint (RSA) and repeated change-of-direction performance (RCOD) remains unclear. Therefore, the aim of this study was to investigate the effects of MF on RSA and RCOD performance in soccer players.

**Methods:**

In a randomized, counterbalanced crossover design, 18 male amateur soccer players completed two experimental sessions, separated by a minimum 48 h washout period: (1) a 45-min Stroop task to induce mental fatigue (MF condition), and (2) watching a 45-min emotionally neutral video as control (CON condition). Following each treatment, participants performed a RSA test and a RCOD test. Measured variables included: Visual Analogue Scale for mental fatigue (VAS-MF), Brunel Mood Scale for fatigue (BRUMS-F), and vigor (BRUMS-V) (assessed pre- and post-treatment); average heart rate (HR_ave_) during the treatment, RSA and ROCD test; Visual Analogue Scale for mental effort (VAS-ME) (assessed post-treatment); and blood lactate (BLA) along with ratings of perceived exertion (RPE) following each performance test.

**Results:**

Pre-treatment VAS-MF and BRUMS-F, HR_ave_ during the treatment did not differ significantly between conditions (*p =* 0.155, 0.429 and 0.262, respectively), post-treatment, the MF condition resulted in significantly higher VAS-MF, VAS-ME and BRUMS-F than control condition (*p* = 0.002, <0.001 and 0.05, respectively). The RSA performance [*p* = 0.885, 0.714 and 0.483 for total time (TT), best time (BT) and fatigue index (FI)] and BLA, RPE during two tests were not significantly affected by MF, however the RCOD performance was significantly reduced in the condition of MF (*p* = 0.006 and 0.007 for TT and BT).

**Conclusion:**

MF did not impair RSA performance but significantly reduced RCOD performance in soccer.

## 1 Introduction

Mental fatigue (MF) is a psychobiological state resulting from prolonged high-intensity cognitive activity, characterized subjectively by feelings of tiredness and a lack of energy (Marcora et al., 2009; Smith et al., 2018). Due to its high prevalence in daily life and its detrimental effects on cognitive and exercise performance (Marcora et al., 2009), MF has become a prominent research focus in competitive sports over the past decade. Extensive studies have explored the impact of MF on athletic performance. Current evidence indicates that MF significantly impairs performance in areas such as aerobic endurance, technique, tactical execution, and decision-making, whereas its effects on anaerobic capacity, maximal voluntary contraction strength, muscular power, and sprint performance appear to be less pronounced (Van Cutsem et al., 2017b; Pageaux and Lepers, 2018; Brown et al., 2020). These findings imply that prolonged, sub-maximal efforts with a substantial aerobic component, as well as tasks involving cognitive or executive functions, are particularly vulnerable to MF. Conversely, short-duration, high-intensity activities predominantly fueled by anaerobic metabolism seem to be more resistant.

Soccer is a sport characterized by high cognitive demands (Badin et al., 2016; Smith et al., 2018). During matches, players must maintain prolonged vigilance and concentration, extract relevant information from a dynamically changing environment—such as the positions of opponents and teammates—and make rapid, accurate decisions under severe time constraints (Smith et al., 2018). This sustained cognitive load makes players particularly susceptible to MF during competition (Smith et al., 2018). Previous studies in soccer have suggested that MF may be a key contributor to the decline in various performance metrics, such as running performance, commonly observed in the later stages of soccer matches (Smith et al., 2015, 2016a, 2016b; Badin et al., 2016). Indeed, recent studies have established links between MF and reductions in match running performance and technical proficiency (Smith et al., 2016a; Liu et al., 2025), providing a novel perspective for optimizing in-match performance.

Repeated-sprint performance (RSA) and repeated-change-of-direction performance (RCOD) refer to the capacity to perform multiple bouts of short-duration maximal sprints and directional changes, respectively (Bishop et al., 2011; Wong et al., 2012). These qualities are critical in soccer and have been shown to positively influence match outcomes (Rampinini et al., 2009; Bishop et al., 2011; Wong et al., 2012). Match analyses indicate that players complete 8.6–13.1 m/min of very-high-intensity running and sprinting (Passos Ramos et al., 2019), along with approximately 727 changes of direction per match (Bloomfield et al., 2007). Similar to other physical performance, both RSA and RCOD demonstrate a decline over the course of a match (Krustrup et al., 2006; Rampinini et al., 2011; Romagnoli et al., 2016). Given that RSA imposes considerable aerobic demand (typically lasting >2 min), and RCOD involves notable aerobic and technical components (Gastin, 2001; Sheppard and Young, 2006; Bishop et al., 2011; Gharbi et al., 2015; Baldi et al., 2017), this performance decrement may also be theoretically linked to MF. Existing studies on MF and RSA/RCOD, however, have yielded inconsistent findings, in part because protocols such as 12 × 20 m shuttle runs or signaled sprints with one turn per repetition inadequately represent both RSA and RCOD performance (Angius et al., 2022; Staiano et al., 2024). Clarifying this relationship could provide clearer practical guidance—such as whether MF-inducing activities (e.g., studying, video gaming) should be avoided prior to the assessment or training RSA and RCOD in soccer.

Therefore, this study aims to investigate the effects of MF on RSA and RCOD in soccer players. We hypothesize that MF will impair performance in both tasks. The findings may provide insights that could inform the design of more effective training and evaluation protocols for these capacities, potentially contributing to match performance optimization.

## 2 Methods

### 2.1 Participants

A statistical power analysis was performed *a priori* using G*Power 3.1 to estimate the required sample size for a within-subjects comparison (difference between two dependent means). The analysis was based on a power (1 − *β*) of 0.8, a significance level (*α*) of 0.05, and an assumed effect size (Cohen’s *d*) of 0.8 (Smith et al., 2016a; Greco et al., 2017; Fortes et al., 2020). To allow for an estimated 20% dropout rate, 22 male amateur soccer players were initially recruited. All participants had no history of color blindness, acute or chronic injuries, diagnosed sleep disorders, or smoking habits.

Participants were recruited from a sports university and consisted of amateur soccer players. During the experiment period, four participants withdrew (two for personal reasons and two due to injury), resulting in 18 individuals who completed all tests. Their anthropometric and training characteristics were as follows: age 20 ± 1 years; height 176 ± 7 cm; body mass 71.0 ± 9.9 kg; BMI 22.83 ± 2.55 kg/m^2^; body fat percentage 17.4 ± 5.2%; and soccer training experience 7 ± 4 years. The 18 participants who completed all tests consisted of six forwards, four midfielders, seven defenders, and one goalkeeper.

All participants provided written informed consent after being fully informed of the experimental procedures and potential risks. To minimize expectation bias, the true aim of the study was not disclosed until all testing was completed. During the study, participants were informed that the research aimed to “investigate the effects of pre-training cognitive activity on physiological and psychological responses during interval running training.” The study protocol was approved by the Ethics Committee of Shanghai University of Sport (Approval No. 10277202112T087).

### 2.2 Study design and procedures

A randomized, counterbalanced crossover design was employed. Each participant completed one familiarization session and two experimental sessions at the laboratory, separated by at least 48 h to allow sufficient recovery from any residual mental and physical fatigue and to ensure a return to baseline (Smith et al., 2016a; Lam et al., 2023, 2024, 2025). To control for circadian effects, both experimental sessions for a given participant were scheduled at the same time of day. The entire protocol was completed within 4 weeks.

The familiarization session was conducted to acquaint participants with all testing procedures, tasks, and equipment thereby minimizing learning effects and physical discomfort in subsequent experimental sessions. The session consisted of the following steps: (1) an detailed explanation of overall testing protocol and instructions on the use of the Visual Analogue Scale (VAS), the Brunel Mood Scale (BRUMS), and the Rating of Perceived Exertion Scale (RPE); (2) a practice period of at least 5 min on the MF induced task while wearing a Polar H10 heart rate monitor (Finland), along with brief familiarization with the measurement tools; (3) a short introduction to and practice trial for both the RSA and RCOD performance tests; and (4) recording of basic anthropometric data (age, height, body weight). At the end of the session, participants received standardized instrucions to adhere to the following guidelines before each subsequent experimental session: (1) obtain at least 8 h of sleep the night prior; (2) abstain from alcohol and nicotine for 24 h, and from caffeine for 12 h, prior to testing; (3) consume fluids exceeding 35 mL/kg of body weight on the day before testing; (4) maintain a consistent diet and aim for similar food intake in the 24 h preceding both sessions, consuming a light snack (e.g., toast, orange juice, banana) approximately 2 h before testing; (5) avoid strenuous physical activity in the 24 h before testing; (6) refrain from cognitively demanding tasks (e.g., video games) on test days, particularly in the 3 h preceding the session; and (7) wear similar clothing to both experimental sessions.

**Figure 1:**
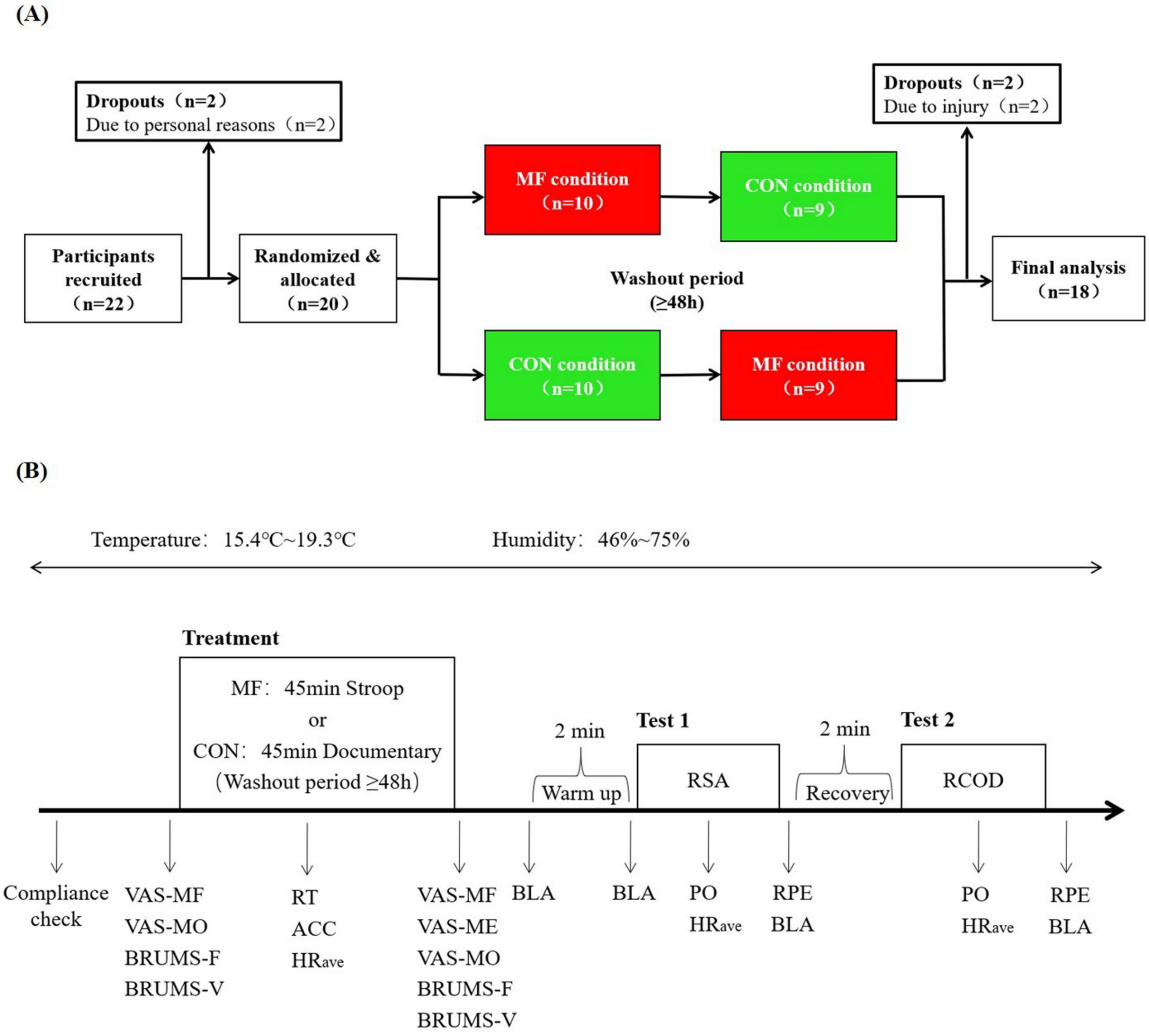
Schematic of the counterbalanced treatment order and the experimental procedure. **(A)** Schematic of the counter-balanced treatment order. **(B)** Flowchart of the experimental procedure. VAS-MF, mental fatigue; VAS-ME, mental effort; VAS-MO, motivation; BRUMS-F, BRUMS fatigue; BRUMS-V, BRUMS vigor; BLA, blood lactate; HR_ave_, average heart rate; RT, response time; ACC, response accuracy; PO, performance outcome; RPE, rating of perceived exertion; RT and ACC only collected in MF condition.

Each experimental session lasted approximately 60 min and followed a standardized procedure: First, participants completed a short questionnaire to confirm adherence to the pre-test instructions. Baseline levels of mental fatigue (VAS-MF), motivation (VAS-MO), and mood [BRUMS fatigue (BRUMS-F) and BRUMS vigor (BRUMS-V)] were then assessed using the respective scales. Subsequently, participants wore the Polar heart rate monitor and completed, in a randomly counterbalanced order determined by an online randomization tool (Figure 1A), either: (1) a 45-min Stroop task to induce MF (MF condition; response accuracy and speed were recorded) or (2) watched a 45-min emotionally neural documentary video as control (CON condition). Immediately following the cognitive task or video, the VAS and BRUMS were readministered to measure VAS-MF, mental effort (VAS-ME), VAS-MO, BRUMS-F, and BRUMS-V, and the average heart rate (HR_ave_) during the task was recorded.

Particpants then performed a standardized 2-min jogging warm-up at an intensity of 60%–70% maximal heart rate (HR_max_), followed by performance testing consisting of the RSA and RCOD tests on an indoor running track. Particpants remained seated for a 2-min rest between tests. The order of tests and the rest interval were implemented according to the protocol adapted from Smith et al. (2016a) and Filipas et al. (2021). Only essential instructions were given during testing; no verbal encouragement was provided. The total duration of physical performance testing after MF induction was approximately 12 min, which falls within the previously reported time window in which MF affects physical performance (Smith et al., 2019; Jacquet et al., 2020). Immediately after each physical test, participants rated their perceived exertion using the RPE scale. Capillary blood samples were drawn from the earlobe to determine blood lactate (BLA) concentration at the following time points: before the warm-up, after the warm-up, after the RSA test, and at 1, 3, 5, 7, and 9 min after the RCOD test. Heart rate was recorded continuously throughout the tests using the Polar monitor. Ambient temperature during testing ranged from 15.4 °C to 19.3 °C, and relative humidity ranged from 46% to 75%. A complete flowchart of the experimental procedure is provided in Figure 1B.

### 2.3 Treatment

#### 2.3.1 MF condition

MF was induced through a 45-min computer-based modified Stroop task (e.g., including both congruent and incongruent trials), adapted from protocols described by Badin et al. (2016) and Habay et al. (2026). The task was programmed and presented using E-Prime 3.0 software. Participants were seated in a quiet, separate room, with one researcher present to ensure adherence to the task. The task involved the sequential, randomized presentation of four Chinese characters for “red,” “green,” “blue,” and “yellow” (displayed in Song typeface, size 57) on a black screen (Lenovo Xiaoxin computer). Each character appeared in one of four possible ink colors: red, green, blue, or yellow. Participants were required to identify the ink color and respond via key press according to a specific rule: for characters displayed in green, blue, or yellow, the response corresponded to the ink color, for characters displayed in red ink, the response corresponded to the semantic meaning of the character itself. Participants first completed 20 practice trials to make sure they understood which key (D for red, F for green, J for blue, and K for yellow) to press for each color. The main task included 1,350 trials to be completed within 45 min. Each trial lasted 2000 ms: a character was displayed for 1,000 ms, followed by a blank screen for 1,000 ms. The color-word matching was set to 50%, meaning that in half of the trials, the meaning of the character matched the color in which it was displayed. Participants were asked to respond as quickly and accurately as possible. If they pressed the wrong key or did not respond within 1,500 ms, the software played a beeping sound to alert them and encourage improved performance.

#### 2.3.2 CON condition

The CON condition was designed to match the duration and setting of the MF condition without induced significant cognitive load followed the protocol used by Badin et al. (2016) and Habay et al. (2026). Participants sat quietly and watched an 45 min emotionally neutral documentary. The documentary *Tale of the Lake* (CCTV6, 2016) was selected. Before the main study, its neutral emotional and cognitive impact was validated in a separate pilot test with 10 participants.

### 2.4 RSA and RCOD tests

**Figure 2:**
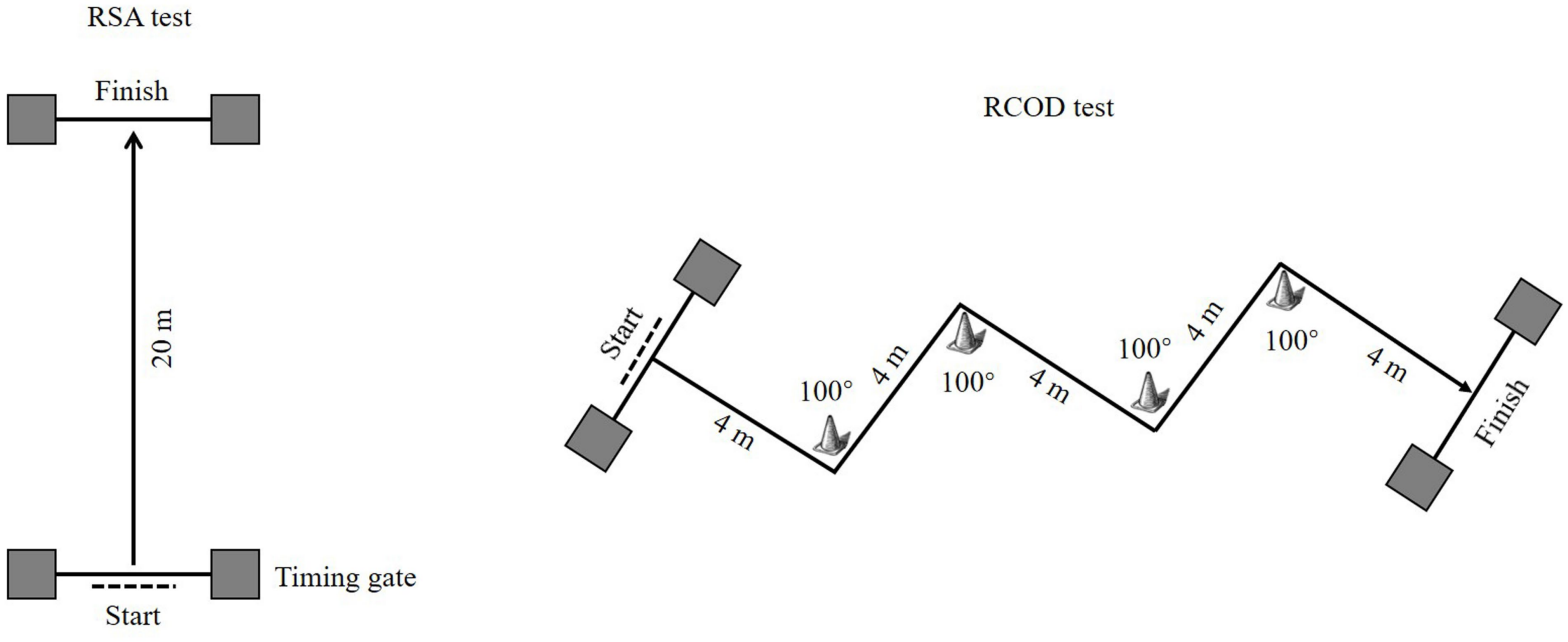
The diagram of RSA and RCOD tests.

Performance in the RSA and RCOD tests was assessed using protocols adapted from Wong et al. (2012, 2015) and Hammami et al. (2017). The RSA test consisted of six maximal 20-m straight-line sprints. The RCOD test consisted of six 20-m sprints, each including four 80° directional changes, with a change executed every 4 m. Before each sprint, participants stood 0.5 m behind the start timing gate (Smartspeed pro, Fusion Sport, Australia). On the “go” signal, they sprinted as fast as possible through the finish gate. After each sprint, a fixed 25-s active recovery period followed, in which participants jogged back to the start line for the next trial. Three performance measures were analyzed: total time (TT), best time (BT), and the fatigue index (FI). FI was calculated according to the formula FI = [TT/(BT × 6)] × 100–100, as reported in references (Glaister et al., 2008; Hammami et al., 2017). Diagrams showing a single trial of the RSA and RCOD tests are provided in Figure 2.

### 2.5 Data collection and measurement

#### 2.5.1 Subjective measures

VAS-MO and VAS-MF were measured before and after the treatment (stroop task or video); VAS-ME was assessed only after the treatment. All three variables were rated using a 100-mm VAS scale, a well-established tool for quantifying motivation, mental fatigue, and mental effort (Veness et al., 2017; Smith et al., 2019). The scale consist of a 100-mm horizontal line anchored on the left with the phrase “Not at all” (representing a complete absence of the state) and on the right with “Extremely” (representing the maximum level), with no intermediate markings. During administration, the researcher asked one of three questions: “How motivated are you to perform the upcoming test?,” “How mentally fatigued do you feel right now?,” or “How much mental effort are you currently exerting?.” Participants then drew a vertical line on a paper version of the scale at the position that best matched their immediate experience. The score for each variable was defined as the distance in millimeters from the left anchor to the participant’s mark. To minimize self-report bias and ensure a common understanding of the term “mental fatigue” a standardized definition, consistent with that used by Marcora et al. (2009) and Smith et al. (2019) was given to all participants prior to the first assessment.

Mood was evaluated before and after the treatment using the Chinese version of BRUMS scale by Zhang et al. (2014). This 23-item instrument comprises six subscales: Anger, Confusion, Depression, Fatigue, Tension, and Vigor. Each item is scored on a 5-point Likert scale (0 = Not at all, 1 = A little, 2 = Moderately, 3 = Quite a bit, 4 = Extremely). For the purposes of this study, only the Fatigue (BRUMS-F) and Vigor (BRUMS-V) subscales were analyzed.

RPE were obtained immediately upon completion of both the RSA and RCOD tests, using the classic 6–20 RPE scale developed by Borg (1998). Each number on the scale corresponds to a descriptor of exertion intensity. Participants were presented with the scale after each test and asked either to point to or verbally state the number that best represented their perceived effort.

#### 2.5.2 Cognitive measures

RT and ACC were collected automatically using E-Prime 3.0 during the Stroop task and exported to Excel for analysis. Performance was summarized by computing the average RT and ACC for every 9 min of the task. RT analyses excluded trials with no response (>1,500 ms) or responses shorter than 200 ms. ACC was analyzed directly from the raw trial data.

#### 2.5.3 Physiological measures

Heart rate was monitored during the cognitive tasks (Stroop/video) and the physical performance tests (RSA and RCOD) using a Polar H10 chest strap (Finland), with data recorded at 1 Hz. The average heart rate (HR_ave_) for each phase was automatically computed by the Polar Beat software for analysis.

Blood lactate (BLA) was measured from 10 μL capillary blood samples drawn from the earlobe before warm-up, after warm-up, immediately after the RSA test, and at 1, 3, 5, 7, and 9 min following the RCOD test. All samples from a given participant were analyzed together after the session using a Biosen_Line analyzer (EKF, Germany) based on the enzyme-electrode method. For analysis, the peak BLA value from the series of measurements taken after the RCOD test was used.

### 2.6 Statistical analysis

Statistical analyses were conducted using SPSS 20.0 (IBM, United States). Data are presented as mean ± standard deviation (*x* ± *s*). The normality of data distribution for all variables was assessed using the Shapiro–Wilk test and visual inspection of histograms. Between-condition comparisons (MF vs. CON) were performed as follows: Paired-samples *t*-tests were used for VAS-MF (pre-, post-treatment, and the change score), VAS-ME (post-treatment), VAS-MO (pre-, post-treatment, and change score), BRUMS-V (pre-, post-treatment, and change score), HR_ave_ during the treatment and physical tests, RPE, and performance of RSA and RCOD. Wilcoxon signed-rank tests were used for BRUMS-F (pre-, post-treatment, and change score). BLA was analyzed with a two-way (condition × time) repeated-measures analysis of variance (ANOVA). Within-group analyses of RT and ACC were conducted with one-way repeated-measures ANOVA. When the assumption of sphericity was violated (for factors with more than two levels), the Greenhouse–Geisser correction was applied. Significant main effects from ANOVA were further examined using Bonferroni-corrected post-hoc pairwise comparisons. All statistical tests were two-tailed, with the significance level set at *α* = 0.05. Effect sizes are reported as: Cohen’s *d* (*d*) for paired *t*-tests, *r* for Wilcoxon tests, and partial eta-squared (*η*^2^*_p_*) for repeated-measures ANOVAs. For significant ANOVA results, *d* or *r* are also provided for post-hoc pairwise comparisons, and 95% confidence intervals (95% CI) are reported for *r* and *d*. The effect sizes was rated as follows: *d* (Smith et al., 2016b): trivial (<0.2), small (0.2–0.6), moderate (0.6–1.2), large (1.2–2.0), very large (>2.0); *r* (Fritz et al., 2012): small (0.1), medium (0.3), large (0.5); and *η*^2^*_p_* (Smith et al., 2019): negligible (<0.04), small (0.04–0.24), medium (0.25–0.63), large (≥0.64).

## 3 Results

### 3.1 MF induction

No significant difference in VAS-MF was found between the MF and CON conditions before the treatment (*t* = 1.487, *p* = 0.155, *d* = −0.350, 95% CI = −0.822 to 0.131). After the treatment, the MF condition showed significantly higher VAS-MF than the CON condition (*t* = 3.574, *p* = 0.002, *d* = 0.842, 95% CI = 0.293 to 1.374). The pre-to-post change in VAS-MF was also significantly greater in the MF condition (*t* = 4.770, *p* < 0.001, *d* = 1.124, 95% CI = 0.519 to 1.790). Additionally, VAS-ME after the treatment were significantly higher in the MF condition (*t* = 5.520, *p* < 0.001, *d* = 1.301, 95% CI = 0.657 to 1.925) (Table 1).

**Table 1:** Comparison of mental fatigue-related indicators between the MF and CON conditions.

Indicator	Time point	Condition		Test		*d*/*r*	95% CI	
		MF	CON	*t*/*z*	*p*		Lower	Upper
VAS-MF (mm)	Pre-treatment	16.12 ± 11.12	21.21 ± 17.75	1.487	0.155	−0.350	−0.822	0.131
	Post-treatment	51.16 ± 18.22	35.50 ± 21.08	3.574	0.002	0.842	0.293	1.374
	Change	35.04 ± 20.64	14.29 ± 11.98	4.770	<0.001	1.124	0.519	1.709
VAS-ME (mm)	Post-treatment	45.88 ± 20.04	15.08 ± 10.86	5.520	<0.001	1.301	0.657	1.925
BRUMS-F (au)	Pre-treatment	0.94 ± 1.16	1.17 ± 1.29	0.791^a^	0.429	−0.306	−0.798	0.432
	Post-treatment	2.83 ± 2.48	1.72 ± 1.56	1.938^a^	0.050	0.604	0.083	0.866
	Change	1.89 ± 2.19	0.55 ± 1.29	2.207^a^	0.043	0.592	0.105	0.850
BRUMS-V (au)	Pre-treatment	7.44 ± 1.82	6.00 ± 2.00	4.185	0.001	0.987	0.410	1.544
	Post-treatment	5.72 ± 2.94	5.56 ± 2.77	0.338	0.740	0.08	−0.384	0.541
	Change	−1.72 ± 1.56	−0.44 ± 1.79	2.895	0.01	−0.682	−1.189	−0.159
HR_ave_ (bpm)	During treatment	70.78 ± 12.42	68.72 ± 11.55	1.161	0.262	0.274	−0.201	0.741

a Wilcoxon signed-rank test was used.

VAS-MF, mental fatigue; VAS-ME, mental effort; BRUMS-F, BRUMS fatigue; BRUMS-V, BRUMS vigor; HR_ave_, average heart rate.

For BRUMS-F, no significant between-condition difference was observed at baseline (*z* = 0.791, *p* = 0.429, *r* = −0.306, 95% CI = −0.798 to 0.432). Post-treatment, BRUMS-F was significantly higher in the MF condition (*z* = 1.938, *p* = 0.05, *r* = 0.604, 95% CI = 0.083 to 0.866), and the pre-to-post change was also larger in the MF condition (*z* = 2.207, *p* = 0.043, *r* = 0.592, 95% CI = 0.105 to 0.850). Before the treatment, BRUMS-V were significantly higher in the MF condition (*t* = 4.185, *p* = 0.001, *d* = 0.987, 95% CI = 0.410 to 1.544). After the treatment, however, BRUMS-V did not differ between conditions (*t* = 0.338, *p* = 0.740, *d* = 0.08, 95% CI = −0.384 to 0.541). The change in BRUMS-V from pre- to post-treatment was significantly larger in the MF condition (*t* = 2.895, *p* = 0.01, *d* = −0.682, 95% CI = −1.189 to −0.159) (Table 1).

HR_ave_ during the intervention did not differ between conditions (*t* = 1.161, *p* = 0.262, *d* = 0.274, 95% CI = −0.201 to 0.741) (Table 1).

**Figure 3:**
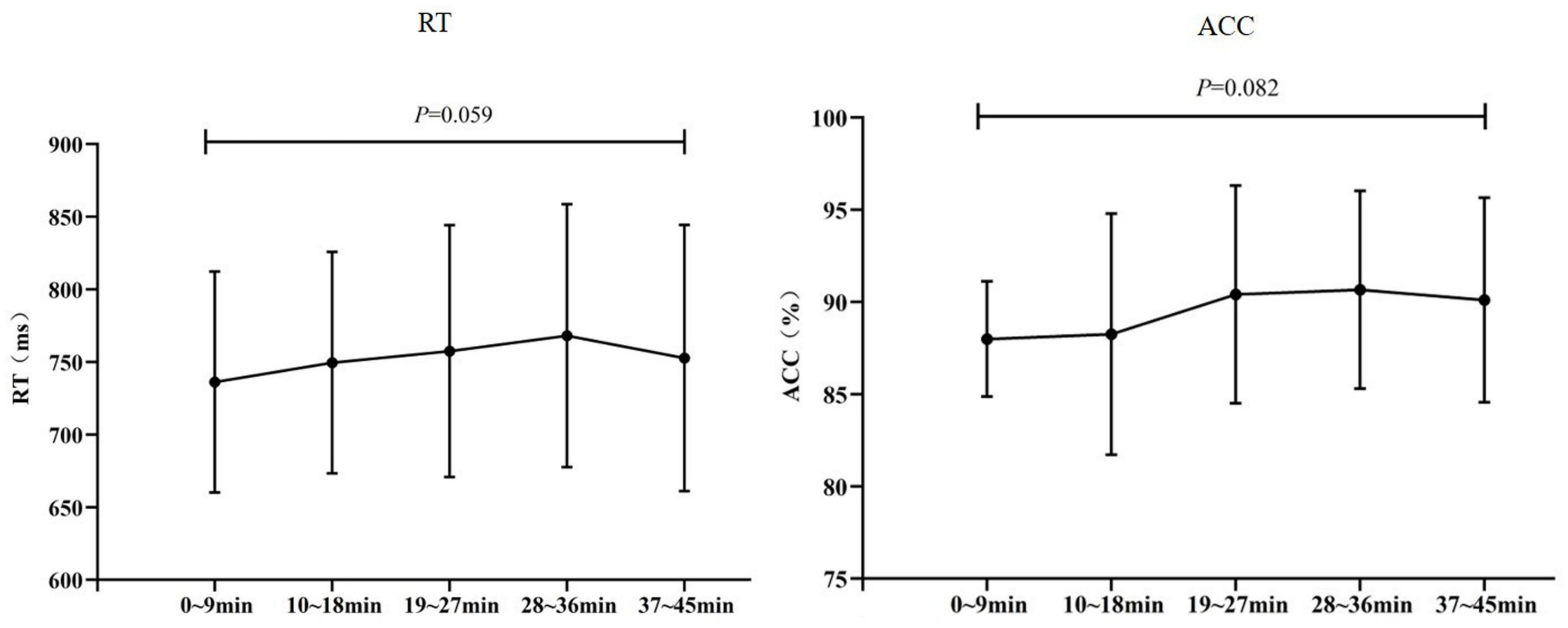
RT and ACC during the stroop task.

Within the MF condition, the main effect of time on RT was not significant (*F*_(2.35,39.98)_ = 2.886, *p* = 0.059, *η*^2^*_p_* = 0.145), nor was the main effect of time on ACC (*F*_(2.62,44.61)_ = 2.464, *p* = 0.082, *η*^2^*_p_* = 0.127) (Figure 3).

### 3.2 RSA and RCOD performance

**Figure 4:**
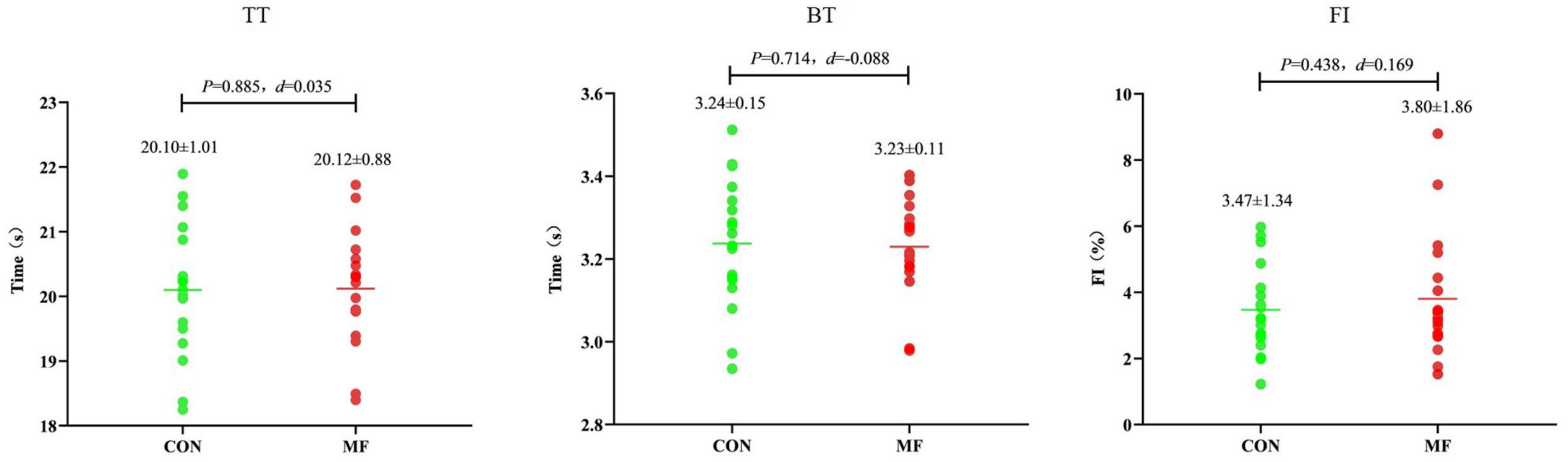
Results of the RSA test.

TT, BT, and FI in the RSA test did not differ significantly between the MF and CON conditions (TT: *t* = 0.147, *p* = 0.885, *d* = 0.035, 95% CI = −0.428 to 0.496; BT: *t* = 0.373, *p* = 0.714, *d* = −0.088, 95% CI = −0.550 to 0.376; FI: *t* = 0.717, *p* = 0.483, *d* = 0.169, 95% CI = −0.299 to 0.632) (Figure 4).

**Figure 5:**
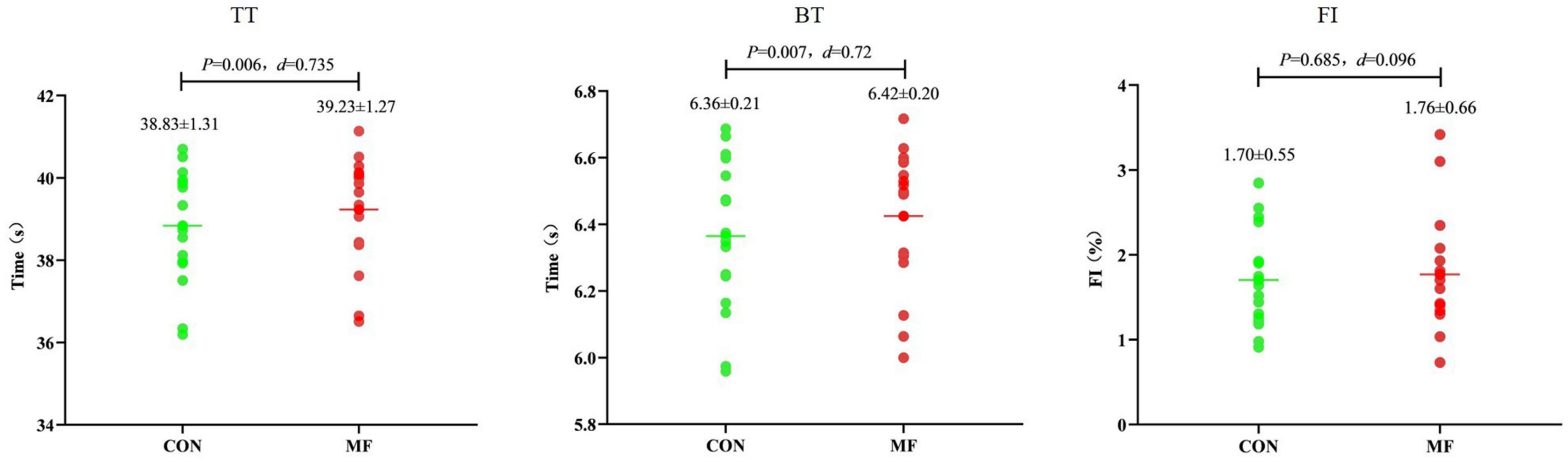
Results of the RCOD test.

In the RCOD test, both TT and BT were significantly greater (i.e., worse) in the MF condition compared to the CON condition (TT: *t* = 3.120, *p* = 0.006, *d* = 0.735, 95% CI = 0.204 to 1.250; BT: *t* = 3.054, *p* = 0.007, *d* = 0.720, 95% CI = 0.191 to 1.232). However, the FI did not differ significantly between conditions (*t* = 0.413, *p* = 0.685, *d* = 0.096, 95% CI = −0.367 to 0.559) (Figure 5).

### 3.3 Other measures

**Figure 6:**
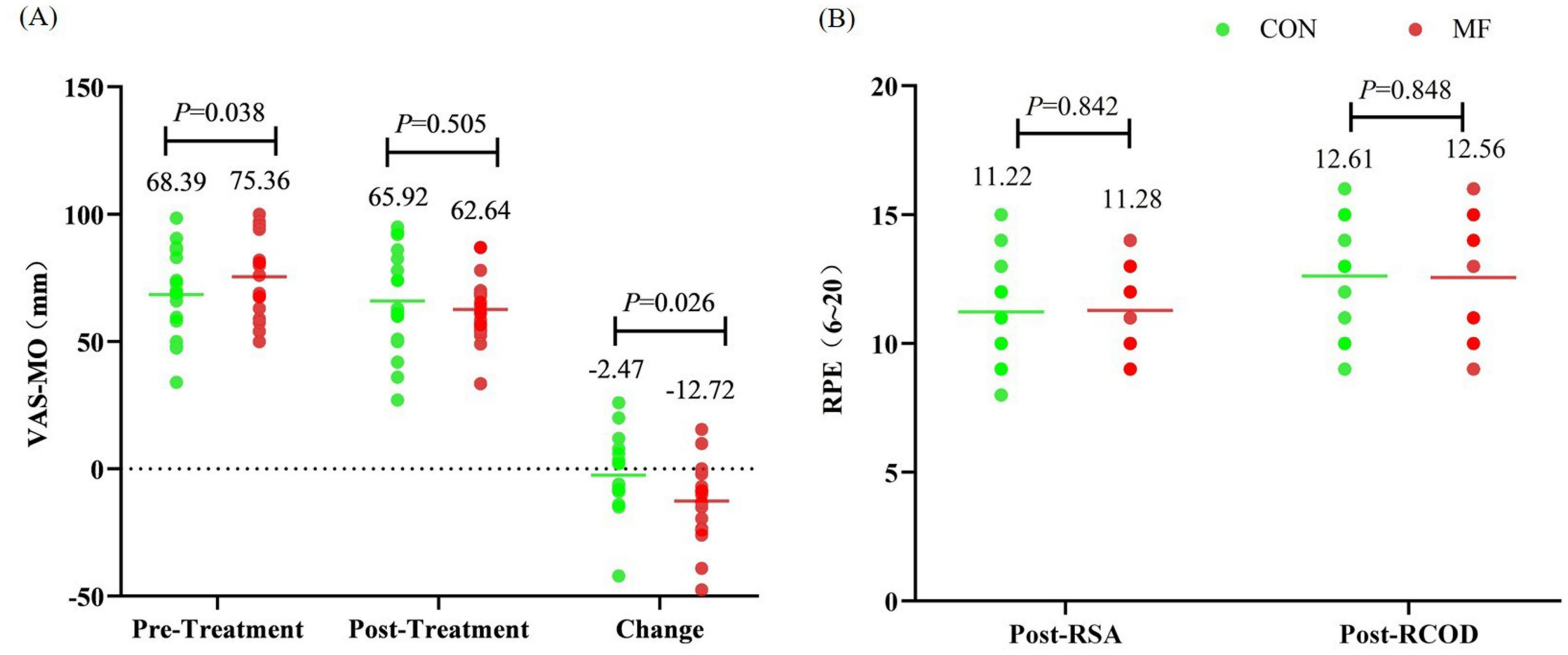
Test results for VAS-MO and RPE. **(A)** VAS-MO, **(B)** RPE.

Before the treatment, VAS-MO was significantly higher in the MF condition compared to the CON condition (*t* = 2.244, *p* = 0.038, *d* = 0.529, 95% CI = 0.028 to 1.017). After the treatment, VAS-MO did not differ between conditions (*t* = 0.681, *p* = 0.505, *d* = −0.160, 95% CI = −0.623 to 0.307). However, the change in VAS-MO from before to after the treatment was significantly greater in the MF condition (*t* = 2.439, *p* = 0.026, *d* = −0.575, 95% CI = −1.068 to −0.067) (Figure 6A).

RPE measured immediately after the RSA and RCOD tests showed no significant differences between conditions (after RSA: *t* = 0.203, *p* = 0.842, *d* = 0.048, 95% CI = −0.415 to 0.509; after RCOD: *t* = 0.195, *p* = 0.848, *d* = −0.046, 95% CI = −0.507 to 0.417) (Figure 6B).

**Figure 7:**
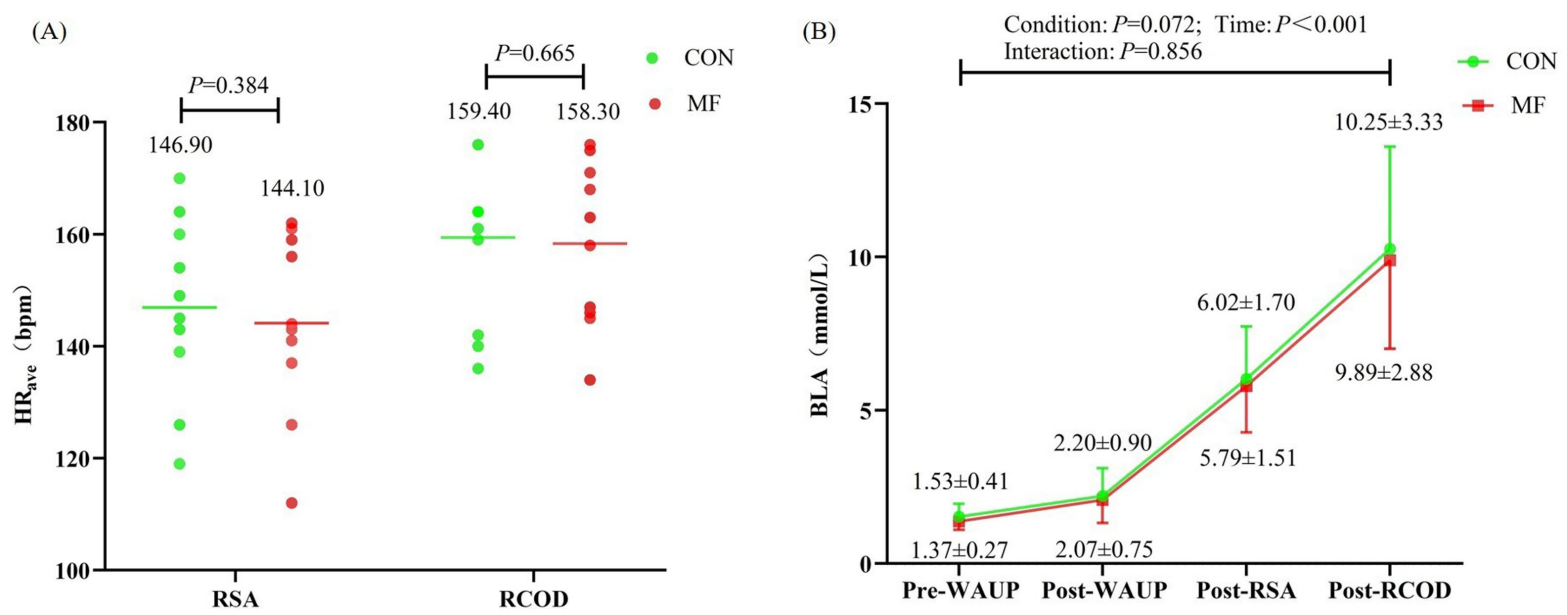
Test results for HR_ave_ and BLA. WAUP, warm up; HR_ave_ was analyzed with a final sample of 10 participants (8 were excluded due to signal transmission failure). **(A)** HR_ave_, **(B)** BLA.

Similarly, HR_ave_ during the physical tests did not differ between the MF and CON conditions (during RSA test: *t* = 0.916, *p* = 0.384, *d* = −0.290, 95% CI = −0.916 to 0.352; during RCOD test: *t* = 0.448, *p* = 0.665, *d* = −0.142, 95% CI = −0.761 to 0.485) (Figure 7A).

For BLA, a two-way repeated-measures ANOVA showed no significant interaction between condition and time (*F*_(1.72,29.31)_ = 0.123, *p* = 0.856, *η*^2^*_p_* = 0.007). The main effect of condition was also not significant (*F*_(1,17)_ = 1.320, *p* = 0.266, *η*^2^*_p_* = 0.072). In contrast, the main effect of time was significant (*F*_(1.26,21.52)_ = 152.50, *p* < 0.001, *η*^2^*_p_* = 0.900), indicating that BLA increased progressively across measurement points. Post-hoc pairwise comparisons with Bonferroni correction confirmed that BLA levels differed significantly between every time point (pre- vs. post-warm-up: *p* = 0.005; all other comparisons: *p* < 0.001) (Figure 7B).

## 4 Discussion

This study aimed to investigate the impact of MF on soccer players’ RSA and RCOD performance. The results indicated that while MF had no significant impact on RSA, it significantly impaired RCOD performance. These findings partially support our hypotheses: they confirm the expected decline in RCOD but do not support the anticipated detrimental effect on RSA.

### 4.1 MF induction

The Stroop task, which requires sustained attention and response inhibition, is a well-established paradigm for inducing MF in sports science including soccer specific research (Badin et al., 2016; Smith et al., 2018). Continuously performing this task for ≥30 min is considered effective for MF induction (Van Cutsem et al., 2017b; Pageaux and Lepers, 2018). Successful induction is typically defined by significant changes—compared to a control condition or pre-task baselines—in one or more domains: subjective, cognitive, or physiological. Among these, significant alterations in subjective measures are the most widely accepted indicators of MF (Van Cutsem et al., 2017b; Pageaux and Lepers, 2018).

In this study, the 45-min Stroop task significantly increased subjective ratings of MF, indicating the successful induction of a perceived state of MF. Before the treatment, the MF and CON conditions showed similar baseline levels of VAS-MF and BRUMS-F. After the treatment, however, the MF condition displayed significantly higher scores than the CON condition on VAS-MF, VAS-ME, and BRUMS-F. The increase from pre- to post-task in VAS-MF and BRUMS-F was also significantly greater in the MF condition, as was the reduction in BRUMS-V. Although not all measures were altered by the MF induction—such as the similar HR_ave_ between conditions, and the absence of clear fluctuation in RT or ACC over the Stroop task—these observations may reflect differences in the sensitivity of various metrics to MF. This interpretation is supported by Smith et al. (2019), who compared three MF-induction protocols in 17 healthy adults. They reported that subjective measures (e.g., BRUMS-F, VAS-MF) were more sensitive to MF than cognitive (RT, ACC) or physiological [electroencephalogram (EEG), heart rate variability (HRV)] measures, with VAS-MF being the most practical and sensitive indicator. The present findings align with other studies (Van Cutsem et al., 2017a; Salam et al., 2018; Filipas et al., 2019). For instance, Filipas et al. (2019) reported that 30 min of Stroop task and 30 min of documentary viewing led to similar HR_ave_ and HRV in cyclists, with stable RT and ACC during the Stroop task. MF was mainly evidenced by changes in subjective mood and workload scales (BRUMS and NASA-TLX). However, an alternative explanation is that the degree of MF induced by the 45-min Stroop task, while sufficient to affect subjective ratings, may not have been strong enough to produce detectable changes in cognitive and physiological measures. In other words, the observed inconsistency across measures could also stem from an insufficient level of MF, rather than solely from differences in metric sensitivity.

Taken together, this evidence suggests that the 45-min Stroop task effectively induced MF in soccer players. Although neurophysiological mechanisms were not directly examined in this study, previous theories have proposed that MF may involve increased prefrontal theta power, altered prefrontal cortex oxygenation, elevated adenosine in the anterior cingulate cortex, and changes in the balance between mental facilitation and inhibition systems (Ishii et al., 2014; Van Cutsem et al., 2017b; Qian et al., 2025).

### 4.2 Effect of MF on RSA and RCOD performance

Based on the work of Bishop et al. (2011)and Wong et al. (2012), RSA is defined as the capacity to maintain a series of short-duration sprints (≤10 s) with very limited recovery intervals (≤60 s). It is a critical performance determinant in team sports including soccer (Rampinini et al., 2009; Bishop et al., 2011). The present study found that MF did not affect soccer players’ RSA. This was reflected in the absence of significant differences between the MF and control conditions in TT, BT or FI during the RSA test. This outcome contrasts with our initial hypothesis. Given that MF impairs aerobic performance (Van Cutsem et al., 2017b; Pageaux and Lepers, 2018) and aerobic capacity contributes to RSA (Rampinini et al., 2009; Chaouachi et al., 2010; Da Silva et al., 2010; Jones et al., 2013; Rodríguez-Fernández et al., 2019), we had anticipated a negative effect.

Several speculative explanations may be considered for this discrepancy, although none were directly tested in the present study. First, studies demonstrating MF-induced aerobic impairment typically employed longer-duration tasks (>5 min) (Van Cutsem et al., 2017b; Pageaux and Lepers, 2018), suggesting the possibility that a minimal exercise duration may be necessary for such effects to emerge. RSA protocols, including the one used here (total duration ≈145 s), are generally shorter (≈2-3 min), which could mean they may be insufficient for MF-related aerobic decrements to manifest. Second, although RSA relies partly on aerobic metabolism (Rampinini et al., 2009; Chaouachi et al., 2010; Da Silva et al., 2010; Jones et al., 2013; Rodríguez-Fernández et al., 2019), the relative contribution of aerobic capacity to RSA performance may be limited. For instance, Aziz et al. (2000) reported that maximal oxygen uptake (VO_2max_) explained only 12% of the variance in TT during an 8 × 40-m RSA test (30-s recovery) in hockey and soccer players. Other studies have similarly found that aerobic metrics account for less than 20% of the variance in RSA outcomes (Chaouachi et al., 2010; Da Silva et al., 2010). Thus, it is possible that the effect of MF on aerobic function may be too small to translate into measurable RSA impairment. This interpretation is not contradicted by our observation that RPE after the RSA test did not differ between conditions, whereas MF-induced aerobic impairment is often accompanied by elevated RPE. Third, despite its aerobic component, RSA is fundamentally a short-duration, high-intensity task dominated by anaerobic metabolism. This view is supported by the elevated BLA concentrations observed here (5-6 mmol·L^−1^ immediately post-test, peaking at 9-10 mmol·L^−1^), which approach levels reported after the classic 30-s Wingate anaerobic test (7–11 mmol·L^−1^) (Jordan et al., 2004). A large body of evidence indicates that short, anaerobic-dominated efforts are largely resistant to MF (Van Cutsem et al., 2017b; Pageaux and Lepers, 2018). One possible neurophysiological explanation, though requiring direct verification, is that the brain regions activated during such efforts (e.g., posterior cingulate cortex) overlap minimally with those implicated in MF (e.g., prefrontal cortex, anterior cingulate cortex, dorsolateral prefrontal cortex) (Smith et al., 2018; Fortes et al., 2024).

Similar to RSA, RCOD is a key performance determinant in soccer, defined as the capacity to maintain directional-change speed during matches or training (Wong et al., 2012, 2015). Although RSA and RCOD protocols are structurally similar, they represent distinct physiological qualities (Wong et al., 2012). Beyond clarifying the RSA–MF relationship, this study examined the effect of MF on RCOD. The results showed that MF impaired RCOD, as indicated by significantly higher TT and BT in the MF condition compared to the control, which aligns with our hypothesis. While the FI did not differ significantly—a discrepancy possibly due to its lower reliability(Wong et al., 2012; Hammami et al., 2018)—the overall pattern indicates a negative MF effect on RCOD.

Like RSA, the RCOD test was short (≈165 s) and highly intensive, and its aerobic contribution may likewise be insufficient to explain the performance decline (supported by similar post-test RPE between conditions). The fact that RCOD was impaired while RSA was not suggests the possibility that MF influences RCOD through pathways beyond aerobic metabolism. Drawing on previous literature, the following hypothetical mechanisms are offered as potential directions for future research, though none were directly measured in this study: (1) Skill factor: Change-of-direction performance, a core component of RCOD, involves a skill element that becomes more pronounced with sharper angles and increased turning frequency (Sheppard and Young, 2006). Since MF has been shown to impair technical/skill performance in soccer (Badin et al., 2016; Smith et al., 2016a), it is plausible that MF may indirectly compromise RCOD via this route. (2) Decision-making factor: Effective RCOD requires continuous decisions about when to accelerate and decelerate. MF is known to impair decision-making in athletes (Smith et al., 2016b; Fortes et al., 2023), which could consequently degrade RCOD. (3) Balance factor: Balance accounts for 25%–75% of the variance in change-of-direction speed among soccer players (Rouissi et al., 2018) and can be negatively affected by MF (Qu et al., 2020), offering another potential pathway for MF to impair RCOD. These hypotheses are partly supported by Veness et al. (2017), who found that MF worsened performance in a change-of-direction-like test (“Run-Two”) in elite cricketers. While some rugby studies have reported no MF effect on change-of-direction performance (Weerakkody et al., 2020), it is possible that discrepancies may arise from differences in sport, athlete level, protocol, or context, as resistance to MF may vary across populations (Martin et al., 2016; Daneshgar-Pironneau et al., 2025). Given the present RSA findings and prior evidence, one might speculate that for soccer players, MF could impairs RCOD through a combined effect on multiple factors—aerobic, technical, decisional, and balance-related—rather than through any single pathway.

### 4.3 Effect of MF on other measures

In addition to measuring RPE and BLA immediately after the RSA and RCOD performance tests, this study also assessed participants’ motivation to perform these tasks and recorded HR_ave_ during the tests. The results showed that, under MF, athletes’ motivation to undertake the RSA and RCOD tests, along with their HR_ave_ during the tests and post-test RPE and BLA values, did not differ significantly from those of the control condition. These findings appear broadly consistent with the previous literature (Van Cutsem et al., 2017b; Pageaux and Lepers, 2018), which suggests that MF may have little influence on athletes’ motivation to engage in physical tasks or on physiological markers measured during the tasks. Furthermore, the RPE data may suggest that MF had a limited effect on the aerobic component of performance in both RSA and RCOD tasks, although it should be noted that previous research has often reported that when MF negatively affects aerobic endurance performance, it is typically accompanied by elevated RPE (Smith et al., 2016a, 2018; Filipas et al., 2021).

### 4.4 Limitation

Several limitations should be acknowledged. First, the participants were male amateur college soccer players; therefore, whether the findings can be extended to female, elite, or youth players remains uncertain. Second, the findings are derived from a controlled laboratory setting, and their direct applicability to real-world matches or training remains to be verified. Third, MF was induced only via a 45-min Stroop task, and physical performance was measured solely with a 6 × 20-m (25-s recovery) protocol. Whether results would hold with longer induction times, other cognitive tasks, or different performance tests is unknown. Finally, only the effect of acute MF was tested. Future studies should explore the impact of accumulated or chronic MF on RSA and RCOD in soccer players.

## 5 Conclusion

MF had no effect on RSA performance but had a detrimental effect on RCOD performance. These findings tentatively suggest that declines in RCOD performance might be linked to MF. Consequently, in the specific context of RCOD assessment or experimental protocols, practitioners may consider standardizing or controlling for prior cognitively demanding tasks to reduce measurement variability. Any broader application to pre-competition behavior or match performance remains speculative and requires further ecologically valid investigation.

## Data Availability

The raw data supporting the conclusions of this article will be made available by the authors, without undue reservation.
